# Tenon Capsule Grafting versus Autologous Scleral Graft in Ahmed Glaucoma Valve Surgery

**DOI:** 10.1155/2020/1248023

**Published:** 2020-05-24

**Authors:** Faried M. Wagdy

**Affiliations:** Ophthalmology Department, Faculty of Medicine, Menoufia University, Shebeen El-Kom, Egypt

## Abstract

**Objective:**

To compare between the surgical outcomes of Tenon capsule grafting and autologous scleral graft in Ahmed glaucoma valve (AGV) surgery in the management of refractory glaucoma and prevention of tube exposure. *Patients and Methods*. This prospective randomised study included 30 eyes of 30 patients with refractory glaucoma, who were aged between 46 and 58 years and diagnosed with refractory glaucoma. This study was conducted in Menofia University Hospital between July 2018 and December 2019. Informed patient consent was obtained. The studied eyes were divided into two groups: the first group included 15 eyes for which AGV with Tenon capsule grafting was performed, while the second group included 15 eyes for which AGV with autologous scleral graft was performed. All patients were followed up for one year after the surgery. The outcomes were evaluated according to intraocular pressure (IOP) and the number of postoperative glaucoma medications. Visual acuity, visual field, number of postoperative glaucoma medications, and postoperative complications were followed throughout the 1-year follow-up period.

**Results:**

There was a significant reduction in IOP in both groups, with more reduction in the Tenon graft group where the mean IOP after one year was 11.66 ± 0.89 mmHg, whereas in the scleral graft group, the mean IOP was 14.20 ± 4.0 mmHg (*p* value < 0.001). However, the difference between the 2 groups in lowering IOP was insignificant. Regarding postoperative complications, tube exposure was observed in one case in the scleral graft group with associated scleral melting and hypotony, postoperative hypotony was more in the scleral graft group with 3 cases (20%), and in the Tenon graft group, hypotony occurred only in 1 case (6.67%). In addition, less vascular blebs were seen in most cases in the Tenon graft group, while most blebs seen in the scleral graft group were vascular blebs. In addition, both groups showed stability in terms of visual acuity and visual field.

**Conclusion:**

Tenon capsule grafting and autologous scleral grafting might be effective and safe techniques when applied with AGV in the management of refractory glaucoma. Tenon capsule resection with grafting showed relatively low incidence of tube exposure and hypertensive phase.

## 1. Introduction

AGV is a shunt device that is used in refractory glaucoma either primarily or after the failure of conventional procedures. It directs the flow of aqueous through the silicone tube [1]. Hypotony, shallow anterior chamber, corneal-lenticular touch, choroidal detachment, hyphema, erosion and exposure of the tube or plate, extrusion of the implant, and endophthalmitis are usual complications associated with Ahmed glaucoma valve (AGV) [2]. Tube erosion almost always occurs at the proximal limbal conjunctiva if left uncovered, leading to exposure and an increased risk of endophthalmitis [3]. There are two common surgical methods of tube coverage used in AGV surgery: the preserved donor sclera and the autologous scleral flap [4]. Grafts, however, prevent tube exposure, carry the risk of scleral melting, and place high financial burden; thus, it is not an excellent surgical technique to prevent AGV tube exposure [5, 6]. Tenon capsule used as in a recent technique completely buries the tube within the sclera by using a cover of a long scleral flap with Tenon advancement and duplication to avoid tube exposure [7]. Also, partial intraoperative Tenon's capsule resection with the use of adjunctive of mitomycin C is effective in developing thin, avascular blebs in eyes undergoing Ahmed glaucoma valve insertion [8].

This study aimed to clarify the difference in surgical outcomes between autologous scleral grafts and Tenon duplicated grafts in the prevention of AGV tube exposure in patients with refractory glaucoma.

## 2. Patients and Methods

This prospective randomized study that used a computer-generated random number table included 30 eyes of 30 patients aged between 46 and 58 years and diagnosed with refractory glaucoma. These patients presented with elevated intraocular pressure (IOP) despite maximal tolerated medical treatment after previous trabeculectomy surgery. This study was conducted in Menofia University Hospital between July 2018 and December 2019. Approval from the Ethical Scientific Committee was obtained. The studied eyes were divided into two groups: the first group included 15 eyes for which AGV with Tenon capsule grafting was performed, while the second group included 15 eyes for which AGV with autologous scleral graft was performed. All patients were followed up for one year after the surgery. The outcomes were evaluated according to intraocular pressure (IOP) and the number of postoperative glaucoma medications. Complete success in IOP was considered as IOP less than 21 mmHg without treatment, a qualified success in IOP was considered as IOP less than 21 mmHg with medical treatment, and a failure in IOP control was considered if IOP was more than or equal to 20 mmHg after surgery. Hypotony was defined as an IOP < 6 mmHg. Visual acuity, visual field, number of postoperative glaucoma medications, and postoperative complications were followed throughout the 1-year follow-up period.

Preoperative examination for all patients included baseline preoperative IOP using the Goldmann applanation tonometer, VA using the Snellen E chart, VF using the Humphrey visual field analyser, angle examination using the goniolens, optic disc examination using the Volk + 90 lens, and the slit lamp examination for the assessment of corneal clarity and anterior chamber depth. Type of glaucoma in each patient and preoperative antiglaucomatous medications were clarified (Table 1).

### 2.1. Peribulbar Anaesthesia Was Used for All Patients

#### 2.1.1. Operative Technique in Group I

**Figure 1:**
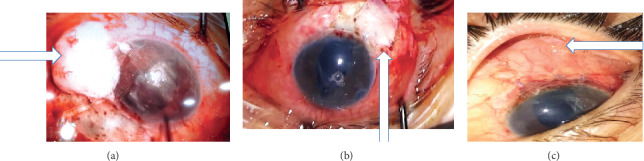
(a) Tenon capsule after resection (as seen by the blue arrow). (b) Tenon capsule duplicated and sutured to the underlying sclera (as seen by the blue arrow). (c) Thin bleb with minimal vascularization (3 months after surgery) (as seen by the blue arrow).

The superiortemporal conjunctiva was incised and appropriately cauterised, mitomycin C (MMC) was applied by using 3 sponges soaked with MMC with a concentration of 0.2 mg/ml which were placed deeply over the sclera, left for 2 minutes and followed by irrigation using balanced salt solution, and the valve body plate was placed approximately 10 mm posterior to the corneal limbus. The tube was ligated with an 8-0 vicryl with the underlying sclera. The Tenon capsule was dissected and resected up to the posterior limit of the AGV body. The double layer of the Tenon capsule was fashioned with a diameter 8 mm long and 5 mm wide extended anteriorly to the limbus and sutured tightly using 8-0 vicryl sutures (6 sutures, 3 on either side) into the underlying sclera (Figure 1).

#### 2.1.2. Operative Technique in Group II

**Figure 2:**
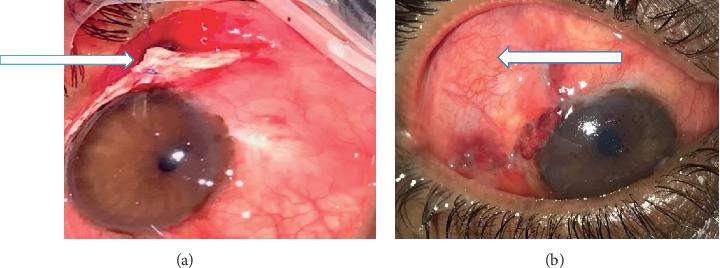
(a) Autologous scleral graft sutured to the underlying sclera (as seen by the blue arrow). (b) Vascularised bleb with the tube in the anterior chamber (3 months after surgery) (as seen by the blue arrow).

It was the same as that used in group I with the difference that the Tenon capsule is not resected and an autologous scleral graft from the upper superonasal sclera of the same eye was taken (8 mm long and 5 mm wide extended anteriorly to the limbus and sutured tightly using 8-0 vicryl suture (6 sutures, 3 on either side) into the underlying sclera) (Figure 2).

Postoperative treatment included combined antibiotic and steroid eye drops every four hours in the first week with gradual tapering after 2 weeks.

The follow-up criteria for these patients included IOP, VA, VF, and optic disc examination using the Volk + 90 lens and slit lamp examination including bleb examination for its shape and vascularity, and any AGV tube corneal or lenticular touch, tube erosion, or exposure. Those who required additional glaucoma surgery, implant removal, or who had phthisis bulbi were considered failures.

### 2.2. Statistical Analyses

Results were statistically analysed by SPSS version 22 (SPSS Inc., Chicago, IL, USA). Independent *t-* and paired *t*-tests were used for parametric data. Mann–Whitney and Wilcoxon signed rank tests were used for nonparametric data. Fisher's exact test was used for qualitative variables. Pearson correlation was used to detect strength and association between pre- and postoperative variables. *p* value was considered significant if <0.05.

## 3. Results

**Figure 3:**
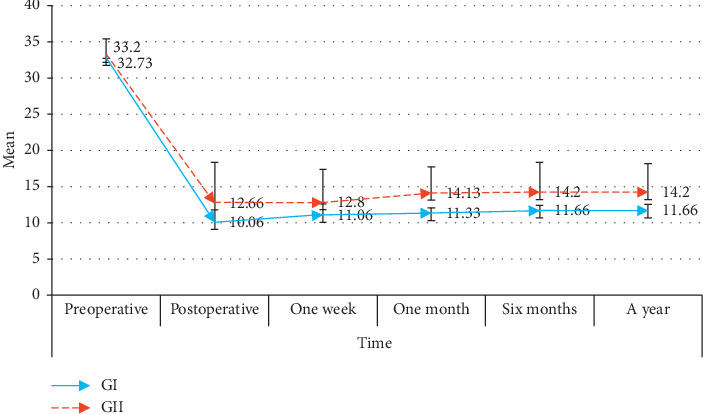
Follow-up of the studied IOP among the studied groups over time.

**Figure 4:**
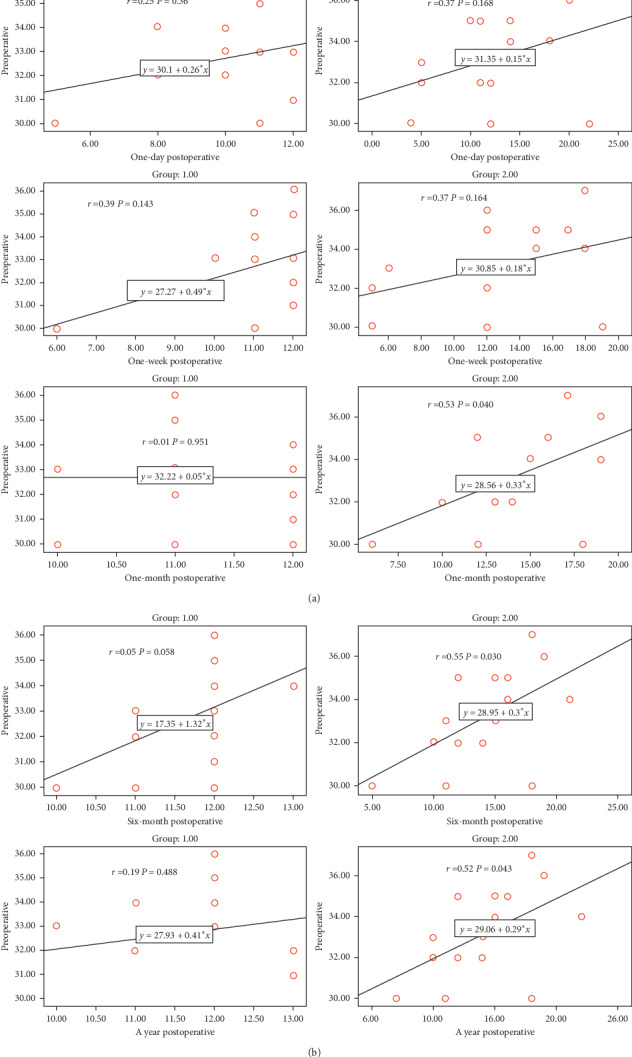
Preoperative IOP versus postoperative IOP values at different follow-up visits.

This study analysed the cases of 30 patients with refractory glaucoma despite maximally tolerated medical treatment and previous trabeculectomy with mitomycin C: 18 males and 12 females. The baseline patient characteristics are described in Table 1 with no statistically difference between the two groups. The preoperative mean IOP was 31.73 ± 1.90 mmHg (range 30–35 mmHg) in group I and 32.20 ± 2.21 mmHg in group II (range 31–36 mmHg). IOP showed a significant reduction in both groups throughout the follow-up period. The mean IOP in group I after 1 year was 11.66 ± 0.89 mmHg (*p* < 0.001), while in group II, the mean IOP was 14.20 ± 4.0 mmHg (*p* < 0.001) (Table 2 and Figure 3). All of these parameters for the follow-up period did not differ significantly between the two groups. Preoperative IOP versus postoperative IOP values in each group were studied, and there was no significant correlation between pre-post IOP in group I while in group II there was significant positive correlation between preoperative IOP and postoperative IOP (1 month, 6 months, and a year) (*p* < 0.05) (Figure 4).

Cutoff postoperative IOP values were different between both groups, and postoperative IOP values that were <14 mmHg in group I occurred in 14 eyes (93.3%) while in group II, 10 cases (66.7%) were below that IOP level, and patients in the Tenon group have lower final IOP which may be due to less fibroblastic proliferation that usually occurred in conventional AGV surgery. However, there was a significant reduction in IOP in both groups: hypertensive phase occurred in 1 case (6.67%) in group I, which showed an increase in IOP after 3 weeks. The IOP was 25 mmHg and declined to 12 mmHg with 2 medications in the form of prostaglandin analogue (PGA) and beta-blocker topical medications. In group II, 3 cases (20%) showed elevated IOP after 1 month and 2 cases showed improvement in IO with a decline from 27 mmHg to 14 mmHg and 28 mmHg to 13 mmHg, respectively, using 2 medications in the form of PGA and beta-blocker topical medications; the IOP in the third case was 29 mmHg and did not decline despite 3 medications in the form of alpha-agonist and beta-blocker and prostaglandin. This case needed exploration of the valve where severe fibrosis was seen around the tube and the valve body; thus, removal of fibrous tissue followed by reimplantation of the tube was performed. Those cases that did not require further antiglaucoma medications were classified as a complete success; such cases were higher in group I (93.3%) than in group II (80%). The incidence of the hypertensive phase in group I was lower than in group II which reflected that the postoperative fibrosis around the valve plate which usually occurred with conventional AGV was decreased after the Tenon resection and so more antiglaucomatous medications were used in group II to lower IOP.

**Figure 5:**
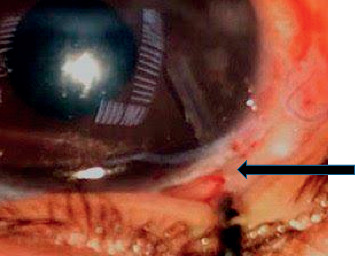
Scleral melting with conjunctival erosion and tube exposure (as seen by the arrow) after 3 months of AGV with autologous scleral graft in the female patient.

Tube exposure was seen in one case (6.67%) in the scleral graft group with associated scleral melting and hypotony (Figure 5), and postoperative hypotony was more in the scleral graft group with 3 cases (20%), two cases occurred in the first postoperative day and improved after 4 days mostly due to overfiltration and one case due to scleral melting that was seen after 3 months with tube exposure and associated with shallow anterior chamber; while in the Tenon graft group, the hypotony occurred only in 1 case (6.67%), mostly related to overfiltration and associated with shallow anterior chamber (Table 3). In addition, less vascular blebs were seen in the Tenon graft group with 12 cases (80%), while most blebs seen in the scleral graft group were high vascular blebs that were present in 13 cases (86.6%), and this most probably due to the Tenon resection that minimized vascularization under the bleb. This reflects the role of Tenon resection in providing limited fibrosis around the AGV body, in addition to the benefit of fibroblastic proliferation that may occur around the proximal end of the tube, and limits any leakage that may occur during the surgery, otherwise the perfect external tamponade.

The other postoperative complications of the two groups are summarised in Table 3. The anterior chamber was clinically assessed under slit-lamp examination and was defined as iridocorneal touch in the periphery, which was seen in 2 cases only in group I (13.3%) and 4 cases in group II (26.7%) that were associated with hypotony and improved after 2 days with topical steroid and cycloplegic eye drops. Hyphema occurred in one case in group I (6.67%). There was more stability in BCVA and VF in group I, and no cases in group I showed deterioration in visual field or BCVA while 3 cases in group II showed this deterioration, 2 cases presented with increased IOP and showed one-line decline in Landolt's broken ring chart and a more deteriorated visual field; the third case with choroidal detachment showed a decline of 2 lines in Landolt's broken ring chart with deteriorated visual field.

## 4. Discussion

AGV implantation is an effective surgery for reducing IOP in refractory glaucoma [9]. Postoperative complications have been observed with AGV; however, it has a high efficacy in controlling IOP, including tube exposure through conjunctival erosion that may cause major complications such as hypotony, phthisis, and endophthalmitis [10, 11]. Although there are different techniques aimed at preventing tube exposure, such as placement of a patch graft (e.g., fascia lata, pericardium, or donor sclera), there is no ideal technique for preventing exposure of the AGV tube [12].

The aim of this study was to compare between the surgical outcomes of Tenon capsule grafting and autologous scleral graft in AGV surgery to prevent tube exposure and associated complications besides their role in controlling IOP.

This study analysed the cases of 30 patients with refractory glaucoma despite maximally tolerated medical treatment and previous trabeculectomy with mitomycin C. The preoperative median IOP was 31.73 ± 1.90 mmHg (range 30–35 mmHg) in group I and 32.20 ± 2.21 mmHg in group II (range 31–36 mmHg). IOP showed a significant reduction in both groups throughout the follow-up period after 1 year. The median IOP in group I after 1 year was 11.66 ± 0.89 mmHg (*p* < 0.001), while in group II, the median IOP was 14.20 ± 4.0 mmHg (*p* < 0.001). All of these parameters for the follow-up period did not differ significantly between the two groups.

Tube exposure was seen in one case (6.67%) in the scleral graft group, with associated scleral melting and hypotony. Postoperative hypotony was more common in the scleral graft group, which occurred in 3 cases (20%), while in the Tenon graft group, hypotony occurred only in 1 case (6.67%). In addition, fewer vascular blebs were seen in the Tenon graft group with 12 cases (80%), while most blebs seen in the group scleral graft were high vascular blebs that were present in 13 cases (86.6%). This reflects the role of Tenon resection in providing limited fibrosis around the AGV body, in addition to the benefit of fibroblastic proliferation that may occur around the proximal end of the tube, and limits any leakage that may occur during the surgery, otherwise the perfect external tamponade.

The other postoperative complication of the two groups is that the shallow anterior chamber was clinically assessed under slit-lamp examination and was defined as iridocorneal touch in the periphery as seen in 2 cases only in group I (13.3%) and 4 cases in group II (26.7%), which were associated with hypotony and improved after 2 days with topical steroid and cycloplegic eye drops. Hyphema occurred in group I in one case (6.67%). There was stability in BCVA and VF.

The surgical outcome of the autologous scleral graft in AGV surgery in this study was similar to that of another study aimed to compare the efficacy of an autoscleral free-flap graft versus an autoscleral rotational flap graft in AGV surgery. Twenty seven consecutive patients (27 eyes) received a free-flap graft, and 24 consecutive patients (24 eyes) received a rotational flap graft. The mean follow-up time was 55.6 ± 18.3 months for the former and 24.2 ± 5.0 months for the latter (*p* < 0.0001). Tube exposure occurred in two patients in the free-flap group (8.9%) at 24 and 55 months, while no tube exposure occurred in the other group. Graft thinning without conjunctival erosion was recorded in 15 patients (55%) in the free-flap group and in 7 patients (29.1%) in the rotational flap group [13].

Similar results in another study described the safety and efficacy of fresh, human sclera allografts as a patch graft material in AGV surgery. The average preoperative IOP was 33.2 ± 11.1 mmHg on 4.2 ± 1.3 IOP-lowering agents before AGV surgery. IOP decreased significantly to 14.1 ± 4.7 mmHg (*p* < 0.001) on 1.6 ± 1.2 IOP-lowering agents (*p* < 0.001) after an average follow-up of 18.2 ± 15.4 months. There were no cases of early or late blebitis or endophthalmitis, and there was 1 case of conjunctival erosion and tube/plate exposure (1.6%) occurring 1 month after surgery [14].

The effectiveness of using a scleral graft was reported in a study in which a human donor scleral graft was used. The tube was passed through the scleral tunnel parallel to the corneal limbus and shortened at the desired length, with a significant decrease in the mean IOP. In all cases, the scleral patch was found in place during the follow-up period. No tube exposure over the AGV tube or endophthalmitis was seen during the follow-up period [15].

This study was in agreement with another study done to evaluate the effect of supra-Tenon capsule implantation of AGV to decrease the fibrotic potential of the Tenon capsule on bleb formation and its associated effect on IOP control in children with refractory glaucoma. There were 12 eyes (54.6%) with refractory congenital glaucoma; there was a statistically significant difference between the mean preoperative IOP (30.7 ± 2.88 mmHg) and the mean postoperative IOP (16.1 ± 3.60 mmHg). The difference between the mean number of antiglaucoma medications before surgery (1.86 ± 0.4) and after surgery (1.0 ± 0.9) was also statistically significant. Postoperative complications included tube exposure and slippage (10%), hypotony (10%), and hyphema (5%) [16].

The difference between this study and other previous studies are clarified and summarized in Table 4.

This study described that Tenon capsule grafting and autologous scleral grafting are effective and safe techniques when applied with AGV in the management of refractory glaucoma. Tenon capsule resection with grafting provided fewer complications regarding tube exposure and hypotony with less hypertensive phase. This study clarified the advantage of Tenon capsule resection with grafting as a nontime-consuming rapid surgery with no additional materials and low cost in management of refractory glaucoma by AGV; the only disadvantage is sometimes dissection is difficult if there were presence of cicatricial tissues due to previous surgery.

## 5. Strengths and Limitations

The limitations of this study were a relatively small number of patients and short-term follow-up. Long-term ocular surface changes also can be studied in the future like other studies that clarified the long-term ocular surface changes induced by various treatment choices in POAG patients describing the potential of the Xen 45 Gel Stent in preserving ocular surface integrity and the potentiality of improved outcomes for any subsequent surgeries [17]. In addition, more study for morphological changes will be important like the study which clarified the role of intraoperative real-time image-guided technique using intraoperative ultrasound biomicroscopy and optical coherence tomography and represented that it will be the future, even if it still needs more efforts to make the instrumentation functional and simple to use [18]. Strengths of the study were the effectiveness of two methods of tube grafting in AGV surgery that were with low cost, not time consuming, and easily dissected in absence of cicatrising tissues of previous surgeries. The Tenon graft technique provided more good results in this study in spite of the short-term follow-up. In the near future, I plan to work on a large sample with long-term follow-up to detect the degree of effectiveness and would certainly benefit surgeons, especially if they have problems with the supply of other materials to cover the tubes.

## 6. Conclusions

In conclusion, both techniques might be effective in AGV surgery for management of refractory glaucoma; however, Tenon capsule resection with grafting showed relatively low incidence of tube exposure and hypertensive phase. Both techniques provided low cost which is beneficial mainly in developing countries.

## Figures and Tables

**Table 1:** Baseline characteristics of the two groups.

	Groups				*p* value
	Group A *n* = 15 eyes (15 patients)		Group B *n* = 15 eyes (15 patients)		
	No	%	No	%	
Age (mean ± SD)	49.44 ± 3.62		49.25 ± 4.45		0.898
Gender					
Male	10	66.7	8	53.3	0.495
Female	5	33.3	7	46.7	
Preoperative baseline IOP (mean ± SD)	31.73 ± 1.90		32.20 ± 2.21		0.456
Type of glaucoma					
Aphakic glaucoma	4	26.7	5	33.3	
Pseudophakic glaucoma	6	40	4	26.7	0.848
Psedoexfoliation glaucoma	2	13.3	3	20.0	
Pigmentary glaucoma	1	6.7	2	13.3	
Traumatic angle recession glaucoma	2	13.3	1	6.7	
Preoperative medications					
Topical (beta-blocker + CAI + prostaglandin analogue)	10	66.7	9	60	0.704
Topical (beta-blocker + CAI + brimonidine)	5	33.3	6	40	

**Table 2:** IOP of the studied groups.

	IOP					
	Preoperative	Postoperative 1 day	Postoperative 1 week	Postoperative 1 month	Postoperative 6 months	Postoperative 1 year
	Mean ± SD	Mean ± SD	Mean ± SD	Mean ± SD	Mean ± SD	Mean ± SD
GI (no. = 15)	31.73 ± 1.90	10.06 ± 1.83	11.06 ± 1.53	11.33 ± 0.72	11.66 ± 0.72	11.66 ± 0.89
*p* value	—	<0.001^*∗*^	<0.001^*∗*^	<0.001^*∗*^	<0.001^*∗*^	<0.001^*∗*^
GII (no. = 15)	32.20 ± 2.21	12.66 ± 5.67 *M* = 12 IQR = 12–16	12.80 ± 4.57 *M* = 14 IQR = 12–16.5	14.13 ± 3.60	14.20 ± 4.12	14.20 ± 4.0
*p* value	—	<0.001^*∗*^	0.001^*∗*^	0.001^*∗*^	<0.001^*∗*^	<0.001^*∗*^
GI vs. II test	0.61	1.75	2.17	2.95	2.34	2.39
*p* value	0.541	0.080	0.030^*∗*^	0.010^*∗*^	0.034^*∗*^	0.030^*∗*^

IQR: interquartile range; ^*∗*^significant.

**Table 3:** Postoperative complications of the studied groups.

	Groups				Fisher's exact test	*p* value
	GI no. = 15		GII no. = 15			
	No	%	No	%		
Hypotony	1	6.7	3	20.0	1.15	0.598
Shallow AC	2	13.3	4	26.7	0.33	0.651
Hyphema	1	6.7	1	6.7	—	—
Tube exposure	0	0.0	1	6.7	1.03	1.0
Choroidal detachment	0	0.0	1	6.7	1.03	1.0
Scleral melting	0	0.0	1	6.7	1.03	1.0
Hypertensive phase	1	6.7	3	20.0	1.15	0.598
Continuity of antiglaucoma medications	1	6.7	3	20.0	1.15	0.598
No of medication						
Two	1	6.7	2	13.3	2.98	0.421
Three	0	0.0	1	6.7		
No medications	14	93.3	12	80		

**Table 4:** Rate of tube exposure among other previous studies.

Study	Sample size	Rate of tube exposure (%)	Limitations
Wolf et al. [13]	27 eyes (free-flap autoscleral graft)	8.9	Graft thinning without conjunctival erosion in 55% of free-flap autoscleral graft
Tsoukanas et al. [14]	64 eyes	1.6	The high cost of this graft specially in developing countries
Elhefney et al. [16]	22 eyes	10	Short-term follow-up (18 months) and a relatively small number of cases
The current issue	30 eyes	6.6	Short-term follow-up

## Data Availability

The data used to support the findings of this study are included within the article.
